# Early Recognition of Treatment‐Responsive Rapidly Progressive Dementia: The Modified STAM3^m^P Score

**DOI:** 10.1002/acn3.70434

**Published:** 2026-05-27

**Authors:** R. W. van Steenhoven, N. Satyadev, A. E. M. Bastiaansen, Y. D. Piura, J. Kerstens, Y. S. Crijnen, J. Brenner, T. Brand, T. M. Bienfait, S. Veenbergen, J. M. de Vries, F. J. de Jong, E. G. P. Dopper, P. A. E. Sillevis Smitt, H. Seelaar, G. S. Day, M. J. Titulaer

**Affiliations:** ^1^ Department of Neurology Erasmus University Medical Center Rotterdam the Netherlands; ^2^ Department of Neurology Mayo Clinic in Florida Jacksonville Florida USA; ^3^ Department of Neurology University Hospital Antwerp Antwerp Belgium; ^4^ Laboratory Medical Immunology, Department of Immunology Erasmus University Medical Center Rotterdam Rotterdam the Netherlands

**Keywords:** diagnostic score, rapidly progressive dementia, treatment‐responsive dementia

## Abstract

Early identification of patients with treatment‐responsive rapidly progressive dementia (RPD) is important as early treatment improves outcomes. The STAM_3_P score identifies treatment‐responsive RPD using “high risk” presenting features. We optimized performance by adding a time component (i.e., dementia within 3 months) and validated the modified “STAM3^m^P score” in two independent cohorts (*n* = 302). A STAM3^m^P score ≥ 1 identified 96% of patients with treatment‐responsive RPD; ≥ 3 features were highly specific (98%). These data suggest that ≥ 1 STAM3^m^P features effectively identifies treatment‐responsive RPD and may be used to prioritize investigations, whereas ≥ 3 STAM3^m^P features are highly specific and may inform early treatment decisions.

## 1 Introduction

Rapidly progressive dementia (RPD), defined as the development of dementia within one year or incapacitation within 2‐years of the onset of cognitive symptoms [1, 2, 3], requires a unique diagnostic approach, as underlying causes may be treatment‐responsive [1, 2, 4, 5]. However, the multitude of diagnostic tests and time pressure poses a challenge to clinicians [1]. Addressing this challenge is necessary as early identification of treatment‐responsive causes of RPD allows for early treatment, which is associated with better outcomes [6].

The STAM_3_P score was designed to identify patients with high likelihood of a treatment‐responsive causes of RPD at the first presentation based on the presence of clinically accessible features including seizures, tumor, age < 50‐years, mania, movement disorders, MRI suggestive of autoimmune encephalitis (AE), and CSF pleocytosis (≥ 10 cells/mm^3^) [1]. To date, the performance of this tool has been evaluated in a single U.S.‐based cohort, raising questions concerning generalizability.

We evaluated the performance of the STAM_3_P score in an independent RPD cohort from the Netherlands. We further evaluated the performance of a modified criteria incorporating a time‐based component (i.e., dementia within 3‐months of symptom onset; STAM3^m^P score) and compared performance of the modified STAM3^m^P score to the original STAM_3_P score in our Dutch cohort and the original U.S. cohort.

## 2 Methods

### 2.1 Study Design

The Dutch cohort was comprised of patients aged ≥ 18 years who developed dementia within one year of symptom onset [7], and were enrolled in a prospective multicenter study from 2019 to 2024. Three neurologists reviewed information on patient characteristics, clinical presentation, and ancillary testing. The U.S. RPD cohort was comprised of patients aged ≥ 18 years with RPD—defined as the development of dementia within one year or incapacitation within 2‐years of the onset of cognitive symptoms. Patients were prospectively enrolled within a study of RPD at Washington University in St. Louis (St. Louis, MO, USA) and Mayo Clinic in Florida (Jacksonville, FL, USA) from 2016 to 2022 [1, 5]. In both cohorts, patients with obvious causes of RPD (e.g., stroke, traumatic brain injury, infection) were excluded [1]. Final diagnoses were assigned referencing established diagnostic criteria [7, 8, 9, 10, 11]; causes of RPD were defined as potentially treatment‐responsive or non‐responsive following established methods [1].

Patients or legally authorized representatives provided written informed consent. Study procedures and policies were approved by institutional review boards at local institutions (Washington University in St. Louis, Mayo Clinic, and Erasmus University Medical Center).

### 2.2 Statistical Analysis

R statistical software (v4.3.2; R Core Team 2023) was used for statistical analysis. Pearson Chi‐Squared test or Fisher–Freeman–Halton test were used for group comparisons; two‐sided *p* < 0.05 was considered significant. The STAM_3_P score at first presentation was calculated by assigning one point for each of seizures, disease‐associated tumor, age of onset ≤ 50 years, mania, MRI suggestive of AE, movement disorders, and CSF pleocytosis (≥ 10 cells/mm^3^). The performance of cutoff values ≥ 1, ≥ 2, and ≥ 3 were evaluated [1].

Referencing previous literature that identified faster time to dementia onset as a characteristic of treatment‐responsive RPD [1, 3, 12], dementia within 3 months of symptom onset was evaluated as an additional feature associated with treatment‐responsive RPD in univariable and multivariable logistic regression analyses including STAM_3_P measures. This time‐based criterion was subsequently integrated into a modified STAM3^m^P score, which was evaluated within the Dutch and US RPD cohorts. The diagnostic performance of the original and modified scores was compared using area under curves (AUCs; DeLong test) and accuracy at established cutoffs (McNemar's test).

## 3 Results

### 3.1 Patient Characteristics

The Dutch cohort included 147 patients; median age 67 years (range 36–86 years); 76 (46%) female. Ninety‐five of 147 (65%) patients were diagnosed with a potentially treatment‐responsive cause of RPD (Table 1). The frequency of neurodegenerative diseases was lower in the Dutch (14%) versus US cohort (22%; *p* = 0.041); other diagnoses were represented in similar proportions.

**TABLE 1:** Frequency of etiological diagnoses in patients with rapidly progressive dementia across Dutch and U.S. cohort.

Etiological diagnostic categories	Total (*n* = 302)	Dutch cohort (*n* = 147)	US cohort (*n* = 155)	*P*
**Treatment‐responsive**	181 (60)	95 (65)	86 (55)	0.079
Autoimmune encephalitis	109 (36)	58 (39)	51 (33)	0.14
Inflammatory CNS disorder	28 (9)	13 (9)	15 (10)	0.48
Toxic/metabolic	16 (5)	9 (6)	7 (5)	0.36
Neoplastic	11 (4)	5 (3)	6 (4)	0.54
Primary psychiatric disorder	8 (3)	4 (3)	4 (3)	0.61
Epilepsy (non‐inflammatory)	4 (1)	2 (1)	2 (1)	0.67
Vascular	2 (1)	1 (1)	1 (1)	0.74
CNS infection	3 (1)	3 (2)	0 (0)	0.11
**Non‐responsive**	121 (40)	52 (35)	69 (45)	0.079
Neurodegenerative diseases	54 (18)	20 (14)	34 (22)	0.041
Creutzfeldt‐Jakob disease	42 (14)	17 (12)	25 (16)	0.16
Vascular	15 (5)	8 (5)	7 (5)	0.46
Neoplastic	2 (1)	1 (1)	1 (1)	0.74
Other^a^	8 (3)	6 (4)	2 (1)	0.13

Abbreviation: CNS = central nervous system.

^a^ 6 unknown, 2 leukoencephalopathy. See Tables S3 and S4 for performance in individual cohorts.

### 3.2 Modified STAM3^m^P Score

In the combined cohort, dementia within 3 months of symptom onset was observed more frequently in treatment‐responsive diagnoses (56% vs. 31%, *p* < 0.001, Table S1) and was associated with treatment‐responsiveness in univariate (OR 2.93, 95% CI 1.80–4.76, *p* < 0.001) and multivariable logistic regression analyses (OR 3.09, 95% CI, 1.60–5.97, *p* = 0.001; Table S2). Accordingly, this feature was added to the original STAM_3_P score, creating the modified STAM3^m^P score.

#### 3.2.1 Modified STAM3^m^P Score: Dutch Cohort

Overall performance, measured by AUC, between STAM_3_P and STAM3^m^P, increased from 0.79 to 0.83 (*p* = 0.058, Figure S1A). Accuracy, defined as the proportion of correctly classified cases with treatment‐responsive RPD, increased at the cutoff of ≥ 2 (63% vs. 76%; *p* = 0.016) and ≥ 3 (44% vs. 56%; *p* = 0.036; Table S3). Sensitivity increased significantly across all cutoff values: that is, STAM3^m^P improved detection of patients with potentially treatment‐responsive causes of RPD. The presence of ≥ 1 STAM3^m^P features identified 91/95 (96%) of patients with treatment‐responsive cases, with acceptable specificity (37%; Table S3). Modified STAM3^m^P ≥ 2 and ≥ 3 identified more patients with treatment‐responsive cases compared to STAM_3_P (73% vs. 47%; *p* = 0.001 and 34% vs. 14%; *p* < 0.001).

#### 3.2.2 Modified STAM3^m^P Score: US Cohort and Combined Cohort

The AUCs of the original STAM_3_P and modified STAM3^m^P scores were similar in the U.S. cohort (both 0.88, *p* = 0.65; Figure S1B). However, sensitivity improved at cutoff values of ≥ 2 (62% vs. 83%; *p* < 0.001) and ≥ 3 (26% vs. 44%; *p* < 0.001), whereas accuracy increased at the cutoff value of ≥ 3 (59% vs. 68%; *p* < 0.001; Table S4). Increases in sensitivity came at the cost of decreased specificity, with specificity at cutoff values ≥ 1 decreasing from 62% (STAM_3_P) to 41% (modified STAM3^m^P; *p* < 0.001) and at cutoff values ≥ 2 from 93% to 83% (*p* < 0.001). When the modified STAM3^m^P score was applied to the combined cohort, overall AUC increased from 0.84 to 0.86 (*p* = 0.099, Figure S1C). Accuracy and sensitivity increased at cutoff values ≥ 2 and ≥ 3, at the cost of specificity, which decreased at cutoff values ≥ 1 and ≥ 2 (Table 2).

**TABLE 2:** Performance of the STAM_3_P and modified STAM3^m^P scores (Dutch and US RPD cohorts combined).

	STAM_3_P				STAM3^m^P			
	Treatment‐responsive	Non‐responsive	AUC = 0.84		Treatment‐responsive	Non‐responsive	AUC = 0.86	
**≥ 1**	167	51	218	PPV 77% [73–80]	174	74	248	PPV 70% [67–73]
**< 1**	14	70	84	NPV 83% [75–89]	7	47	54	NPV 87% [76–93]
	181	121	302		181	121	302	
	Sens 92% [87–96]	Spec 58% [49–67]		Accuracy 78% [73–83]	Sens 96% [92–98]	Spec 39%* [30–48]		Accuracy 73% [68–78]
**≥ 2**	98	9	107	PPV 92% [85–95]	140	20	160	PPV 88% [82–91]
**< 2**	83	112	195	NPV 57% [53–61]	41	101	142	NPV 71% [65–77]
	181	121	302		181	121	302	
	Sens 54% [47–62]	Spec 93% [86–97]		Accuracy 69% [64–75]	Sens 77%* [71–83]	Spec 83%* [76–90]		Accuracy 80%*[75–84]
**≥ 3**	35	0	35	PPV 100% [90–100]	70	3	73	PPV 96% [88–99]
**< 3**	146	121	267	NPV 45% [45–47]	111	118	229	NPV 52% [49–54]
	181	121	302		181	121	302	
	Sens 19% [14–26]	Spec 100% [97–100]		Accuracy 52% [46–57]	Sens 39%* [32–46]	Spec 98% [93–99]		Accuracy 62%* [57–68]

Abbreviations: NPV = negative predictive value; PPV = positive predictive value; Sens = sensivity. Spec = specificity.

* Statistically significant difference (*p* < 0.05) between STAM_3_P vs. STAM3^m^P.

**FIGURE 1:**
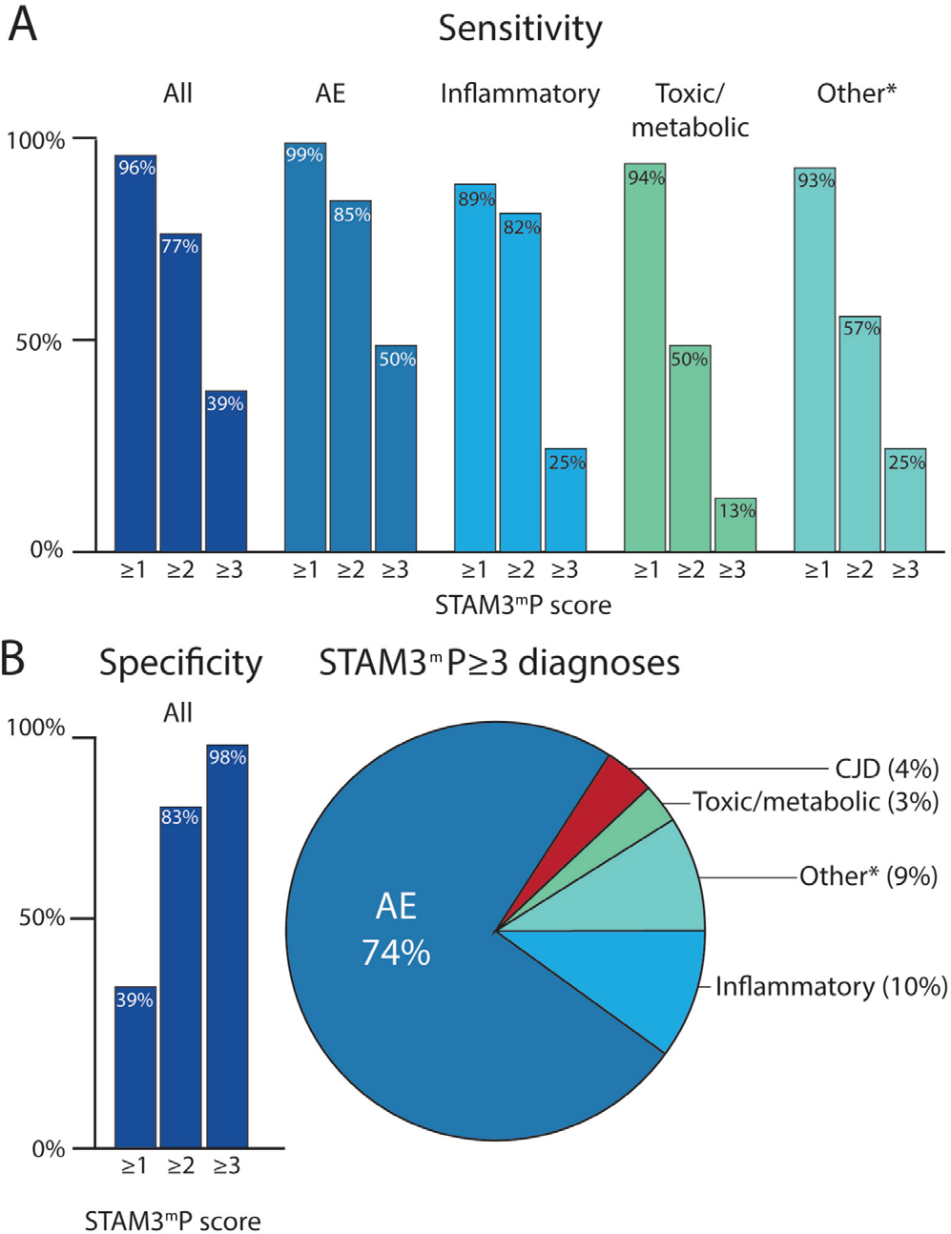
Performance of modified STAM3^m^P score in combined cohort (Dutch and the United States). A. Sensitivity of STAM3^m^P score in different potential treatment‐responsive diagnostic categories. *Other potential treatment‐responsive diagnostic categories: neoplastic, vascular, epilepsy, infectious, and psychiatric. B. Specificity of STAM3^m^P score in total cohort. Pie chart showing potential treatment‐responsive diagnostic categories in patients with ≥ 3 STAM3^m^P features.

The presence of ≥ 1 STAM3^m^P features showed highest sensitivity in patients with RPD attributed to AE (99%), toxic/metabolic causes (94%), and inflammatory central nervous system disorders (89%; Figure 1A, Table S5). Overall, 138/248 (56%) patients with a STAM3^m^P score ≥ 1 met *possible* AE criteria [9], of whom 100 (72%) were diagnosed with AE. Eight additional patients with AE were identified by an elevated STAM3^m^P score and subsequent autoantibody testing (see Table S6 for case examples demonstrating the clinical impact of the STAM3^m^P score).

Of 73 patients with ≥ 3 STAM3^m^P features, 61 (84%) were diagnosed with AE or another inflammatory central nervous system disorder (Figure 1B). Three patients (4%) with CJD had three STAM3^m^P features, including dementia within 3 months (*n* = 3); movement disorders (*n* = 2); age at onset < 50 years (*n* = 1); a newly diagnosed tumor, initially considered disease associated (*n* = 1); seizures (*n* = 1); and CSF pleocytosis (10 cell/mm^3^, no erythrocytes; *n* = 1) that normalized on repeated lumbar puncture.

## 4 Discussion

Time from symptom onset to RPD ≤ 3 months was associated with treatment‐responsive causes of RPD. Accordingly, the addition of this feature to the existing STAM_3_P score improved detection of patients with treatment‐responsive RPD in independent cohorts, with only a modest increase in false positive detection: a modified STAM3^m^P score ≥ 1 recognized 96% of patients with treatment‐responsive RPD, with specificity of 39%. Given the potentially fatal consequences of missed diagnoses [13], we suggest that this sensitivity‐specificity trade‐off is acceptable and advocate that a STAM3^m^P score ≥ 1 may identify RPD patients who require further diagnostic evaluation for treatment‐responsive disorders [14]. Extended diagnostic work‐up should be individualized and may include neuronal autoantibodies testing in serum and CSF, additional neuro‐ and body imaging (e.g., PET), electroencephalography, and, in selected cases, brain biopsy [1, 15, 16]. The clinical AE criteria performed well in this study [9], demonstrating high sensitivity for AE (> 90%). These criteria complement the STAM3^m^P score and may be applied sequentially, with STAM3^m^P score used for initial screening for treatment‐responsive causes of RPD followed by AE criteria to inform next steps in the diagnostic evaluation and treatment of patients with possible AE.

The presence of ≥ 2 and ≥ 3 STAM3^m^P features demonstrated high specificity (83% and 98%) for potentially treatment‐responsive forms of RPD in the combined cohort. Most patients with scores ≥ 3 (~85%) were diagnosed with autoimmune or inflammatory disorders—which typically warrant immunotherapy—suggesting that the modified STAM3^m^P scores ≥ 3 may be used to inform early treatment decisions (e.g., empiric immunotherapy). In RPD patients with ≥ 2 STAM3^m^P features, preliminary treatment may also be considered if an autoimmune/inflammatory cause of RPD is suspected and conditions where immunotherapy may reduce diagnostic yield or cause potentially harmful effects have been reasonably excluded (e.g., central nervous system infections, lymphoma, sarcoidosis) [1, 17, 18]. When considering preliminary treatment for a suspected etiologic diagnosis of AE, clinicians are cautioned to monitor for radiological “red flags” that may identify AE mimics, including persistent enhancement, restricted diffusion and mass effect on MRI [17, 19]. The STAM3^m^P score includes the presence of a disease‐associated tumor. Importantly, if initial tumor screening (e.g., CT scan) is negative, the detection of high‐risk tumor‐associated autoantibodies in blood or CSF still warrants comprehensive tumor screening/surveillance (e.g., repeated FDG‐PET) and, if necessary, oncological treatment [15, 20].

In summary, the presence of ≥ 1 STAM3^m^P features is useful to identify patients with RPD who are likely to benefit from further testing for treatment‐responsive causes, whereas scores ≥ 3 may identify patients who require further testing and may benefit from prompt initiation of immunomodulatory therapies.

## Author Contributions

R.W.S., N.S., G.S.D., and M.J.T. contributed to the conception and design of the study; R.W.S., N.S., A.E.M.B., Y.D.P., J.K., Y.C., J.B., T.B., T.M.B., S.V., J.M.V., F.J.J., E.G.P.D., P.A.E.S.S., H.S., G.S.D., and M.J.T. contributed to acquisition and analysis of data; R.W.S., N.S., G.S.D., and M.J.T. contributed to drafting the text and preparing the figures.

## Funding

The authors have nothing to report.

## Conflicts of Interest

The authors declare no conflicts of interest.

## Supporting information


**Table S1:** Patient characteristics rapidly progressive dementia cohort treatment‐responsive vs. non treatment–responsive diagnoses (Dutch and U.S. cohort combined).
**Table S2:** Univariate and multivariable logistic regression analysis of the STAM3^m^P score (Dutch and US RPD cohort combined).
**Table S3:** Dutch RPD cohort: performance of the STAM_3_P and modified STAM3^m^P scores.
**Table S4:** U.S. RPD cohort: performance of the STAM_3_P and modified STAM3^m^P scores.
**Table S5:** Sensitivity of the modified STAM3^m^P score and possible AE criteria across diagnostic categories (U.S. and Dutch RPD Cohort combined).
**Table S6:** Case examples of treatment‐responsive RPD demonstrating clinical application of the STAM3^m^P score.
**Figure S1:** Comparison of Receiver Operating Characteristic (ROC) Area Under the Curve (AUC) of STAM_3_P and modified STAM3^m^P across different rapidly progressive dementia cohorts (Dutch, the United States, and combined).

## Data Availability

Anonymized study data will be shared pending review of a request to the corresponding author from qualified individuals.

## References

[1] N. Satyadev , P. W. Tipton , Y. Martens , et al., “Improving Early Recognition of Treatment‐Responsive Causes of Rapidly Progressive Dementia: The STAM(3) P Score,” Annals of Neurology 95, no. 2 (2024): 237–248. 37782554 10.1002/ana.26812 PMC10841446

[2] G. S. Day , E. S. Musiek , and J. C. Morris , “Rapidly Progressive Dementia in the Outpatient Clinic: More Than Prions,” Alzheimer Disease and Associated Disorders 32, no. 4 (2018): 291–297. 30222606 10.1097/WAD.0000000000000276 PMC6249048

[3] N. Satyadev , Y. D. Piura , P. W. Tipton , et al., “Standardizing “Rapid”: Applying the Clinical Dementia Rating to Define Rapidly Progressive Dementia,” Neurology 106, no. 1 (2026): e214439. 41397211 10.1212/WNL.0000000000214439 PMC12994857

[4] M. D. Geschwind , H. Shu , A. Haman , J. J. Sejvar , and B. L. Miller , “Rapidly Progressive Dementia,” Annals of Neurology 64, no. 1 (2008): 97–108. 18668637 10.1002/ana.21430 PMC2647859

[5] Y. D. Piura , N. Satyadev , N. Corriveau‐Lecavalier , et al., “Decoding the Clinical Features That Associate With Progression, Causes, and Outcomes in Patients With Suspected Rapidly Progressive Dementia,” Annals of Neurology 98 (2025): 764–776. 40539812 10.1002/ana.27297 PMC12283139

[6] G. S. Day , “Rapidly Progressive Dementia,” Continuum (Minneap Minn) 28, no. 3 (2022): 901–936. 35678409 10.1212/CON.0000000000001089 PMC9580391

[7] G. M. McKhann , D. S. Knopman , H. Chertkow , et al., “The Diagnosis of Dementia due to Alzheimer's Disease: Recommendations From the National Institute on Aging‐Alzheimer's Association Workgroups on Diagnostic Guidelines for Alzheimer's Disease,” Alzheimers Dement 7, no. 3 (2011): 263–269. 21514250 10.1016/j.jalz.2011.03.005 PMC3312024

[8] P. Hermann , B. Appleby , J. P. Brandel , et al., “Biomarkers and Diagnostic Guidelines for Sporadic Creutzfeldt‐Jakob Disease,” Lancet Neurology 20, no. 3 (2021): 235–246. 33609480 10.1016/S1474-4422(20)30477-4 PMC8285036

[9] F. Graus , M. J. Titulaer , R. Balu , et al., “A Clinical Approach to Diagnosis of Autoimmune Encephalitis,” Lancet Neurology 15, no. 4 (2016): 391–404. 26906964 10.1016/S1474-4422(15)00401-9 PMC5066574

[10] C. R. Jack, Jr. , J. S. Andrews , T. G. Beach , et al., “Revised Criteria for Diagnosis and Staging of Alzheimer's Disease: Alzheimer's Association Workgroup,” Alzheimers Dement 20, no. 8 (2024): 5143–5169. 38934362 10.1002/alz.13859 PMC11350039

[11] P. B. Gorelick , A. Scuteri , S. E. Black , et al., “Vascular Contributions to Cognitive Impairment and Dementia: A Statement for Healthcare Professionals From the American Heart Association/American Stroke Association,” Stroke 42, no. 9 (2011): 2672–2713. 21778438 10.1161/STR.0b013e3182299496 PMC3778669

[12] A. E. M. Bastiaansen , R. W. van Steenhoven , M. A. A. M. de Bruijn , et al., “Autoimmune Encephalitis Resembling Dementia Syndromes,” Neurol Neuroimmunol Neuroinflamm 8, no. 5 (2021): e1039. 34341093 10.1212/NXI.0000000000001039 PMC8362342

[13] P. Maat , J. W. de Beukelaar , C. Jansen , et al., “Pathologically Confirmed Autoimmune Encephalitis in Suspected Creutzfeldt‐Jakob Disease,” Neurol Neuroimmunol Neuroinflamm 2, no. 6 (2015): e178. 26601117 10.1212/NXI.0000000000000178 PMC4645173

[14] D. A. Grimes and K. F. Schulz , “Uses and Abuses of Screening Tests,” Lancet 359, no. 9309 (2002): 881–884. 11897304 10.1016/S0140-6736(02)07948-5

[15] M. J. Titulaer , R. Soffietti , J. Dalmau , et al., “Screening for Tumours in Paraneoplastic Syndromes: Report of an EFNS Task Force,” European Journal of Neurology 18, no. 1 (2011): 19–e3. 20880069 10.1111/j.1468-1331.2010.03220.x PMC3086523

[16] R. W. van Steenhoven , S. Salih , J. M. de Vries , et al., “Clinical Impact and Safety of Brain Biopsy in Unexplained Central Nervous System Disorders: A Real‐World Cohort Study,” Annals of Clinical Translational Neurology 12, no. 4 (2025): 792–804. 39981762 10.1002/acn3.70000 PMC12040505

[17] R. W. Van Steenhoven , A. L. Bruijstens , M. Paunovic , et al., “Mimics of Autoimmune Encephalitis: Validation of the 2016 Clinical Autoimmune Encephalitis Criteria,” Neurology Neuroimmunology & Neuroinflammation 10, no. 6 (2023): e200148. 37582614 10.1212/NXI.0000000000200148 PMC10427145

[18] E. P. Flanagan , M. D. Geschwind , A. S. Lopez‐Chiriboga , et al., “Autoimmune Encephalitis Misdiagnosis in Adults,” JAMA Neurology 80, no. 1 (2022): 30–39. 10.1001/jamaneurol.2022.4251 PMC9706400 36441519

[19] M. J. Kelly , E. Grant , A. G. Murchison , et al., “Magnetic Resonance Imaging Characteristics of LGI1‐Antibody and CASPR2‐Antibody Encephalitis,” JAMA Neurology 81, no. 5 (2024): 525–533. 38497971 10.1001/jamaneurol.2024.0126 PMC10949153

[20] F. Graus , A. Vogrig , S. Muñiz‐Castrillo , et al., “Updated Diagnostic Criteria for Paraneoplastic Neurologic Syndromes,” Neurol Neuroimmunol Neuroinflamm 8, no. 4 (2021): e1014. 34006622 10.1212/NXI.0000000000001014 PMC8237398

