# Supplementary material for: Early Recognition of Treatment‐Responsive Rapidly Progressive Dementia: The Modified STAM3mP Score
Source: Ann Clin Transl Neurol. 2026 May 27;13(9):1956–60. doi: 10.1002/acn3.70434 (PMC13394507; doi:10.1002/acn3.70434)
Supplement: Supplementary file 1 [file ACN3-13-1956-s001.docx]

**Supplemental Material**

**List of Supplements: 6 Supplemental Tables and 1 Supplemental Figure**

Supplemental Table 1. Patient characteristics rapidly progressive dementia cohort treatment-responsive vs. non treatment-responsive diagnoses (Dutch and US cohort combined)

Supplemental Table 2. Univariate and multivariable logistic regression analysis of the STAM3^m^P score (Dutch and US RPD cohort combined)

Supplemental Table 3. Performance of the STAM_3_P and modified STAM3^m^P scores (Dutch RPD cohort)

Supplemental Table 4. Performance of the STAM_3_P and modified STAM3^m^P scores (US RPD cohort)

Supplemental Table 5. Sensitivity of the modified STAM3^m^P score and possible AE criteria across diagnostic categories (US and Dutch RPD Cohort combined)

Supplemental Table 6. Case examples of treatment-responsive RPD demonstrating clinical application of the STAM3^m^P score

Supplemental Figure 1. Comparison of Receiver Operating Characteristic (ROC) Area Under the Curve (AUC) of STAM_3_P and STAM3^m^P across different rapidly progressive dementia cohorts (Dutch, US and combined)

**Supplemental Table 1. Patient characteristics rapidly progressive dementia cohort treatment-responsive vs. non treatment-responsive diagnoses (Dutch and US cohort combined)**

|  | **Treatment-responsive**  **N=181 (60%)** | **Non treatment-responsive N=121 (40%)** | **P-value** |
| --- | --- | --- | --- |
| Female sex no. (%) | 83 (46) | 57 (47) | 0.83 |
| Age-at-symptom-onset (median; IQR; range)  Age-at-symptom onset < 50 years no. (%) | 68; 59-74; 22-91  29 (16) | 69; 61-75; 39-88  9 (7) | - **0.019** |
| Initial presentation. (%)  Psychiatric symptoms  Mania  Agitation  Movement disorders  New-onset seizures | 13 (7) 44 (24) 55 (30) 58 (32) | 1 (1) 25 (21) 29 (24) 9 (7) | **0.007** 0.46 0.14 **<0.001** |
| Test findings at presentation   MRI brain of AE^1^  MRI findings of CJD^2^  WBC ≥ 10/µl  Disease-associated tumor no. (%) | 54 (30)  9 (5)  73 (40)  34 (19) | 1 (1)  23 (19)  8 (7)  3 (3) | **<0.001 <0.001**  **<0.001**  **<0.001** |
| Dementia within 3 months no. (%) | 102 (56) | 37 (31) | **<0.001** |

AE=autoimmune encephalitis. CJD=Creutzfeldt-Jakob disease. DWI=diffusion weighted imaging. IQR=interquartile range. CSF=cerebrospinal fluid. ICU=intensive care unit. FLAIR=Fluid-Attenuated inversion recovery. mRS=modified Rankin Scale. PSWC=periodic sharp wave complexes. WBC=white blood cell count. ^1^Unilateral, bilateral, or diffuse T2/FLAIR abnormalities of the medial temporal lobes, supratentorial white matter, basal ganglia, brainstem, and/or cerebellum.[1]

^2^ Diffusion-weighted imaging or T2/FLAIR signal abnormalities in the putamen an caudate or ≥2 cortical regions.[2]

**Supplemental Table 2. Univariate and multivariable logistic regression analysis of the STAM3^m^P score (Dutch and US RPD cohort combined)**

|  | **Univariate** | | | **Multivariable** | | |
| --- | --- | --- | --- | --- | --- | --- |
| **Selected feature** | **OR** | **95% CI** | **P value** | **OR** | **95% CI** | **P value** |
| Seizures at presentation | 5,87 | 2,78-12,39 | <0.001 | 10,95 | 4,43-27,07 | <0.001 |
| Disease-associated tumor | 9,10 | 2,73-30,36 | <0.001 | 13,86 | 3,59-53,48 | <0.001 |
| Age-at-symptom onset < 50 years | 2,37 | 1,08-5,21 | 0.031 | 1,98 | 0,66-5,95 | 0.22 |
| Mania | 9,29 | 1,20-71,94 | 0.033 | 18,10 | 1,87-175,06 | 0.012 |
| Movement abnormalities | 1,39 | 0,82-2,34 | 0.22 | 2,25 | 1,11-4,57 | 0.025 |
| MRI suggestive of AE | 51,02 | 6,95-374,65 | <0.001 | 46,37 | 5,71-376,53 | <0.001 |
| **Dementia within 3 months after symptom onset** | **2,93** | **1,80-4,76** | **<0.001** | **3,09** | **1,60-5,97** | **<0.001** |
| Pleocytosis, ≥ 10 cells/μL in CSF | 9,55 | 4,39-20,75 | <0.001 | 13,17 | 5,39-31,19 | <0.001 |

AE=autoimmune encephalitis. CSF=cerebrospinal fluid. OR=odds ratio. CI=confidence interval.

|  | **STAM_3_P** | | | | **STAM3^m^P** | | | |
| --- | --- | --- | --- | --- | --- | --- | --- | --- |
|  | **Treatment-**  **responsive** | **Not  responsive** | **AUC = 0.79** | | **Treatment-**  **responsive** | **Not  responsive** | **AUC = 0.83** | |
| **≥1** | 85 | 25 | 110 | PPV 77% [72-82] | 91 | 33 | 124 | PPV 73 % [69-77] |
| **<1** | 10 | 27 | 37 | NPV 73% [59-84] | 4 | 19 | 23 | NPV 83 % [63-93] |
|  | 95 | 52 | 147 |  | 95 | 52 | 147 |  |
|  | Sens 89 % [81 - 95] | Spec 52 % [38-66] |  | Accuracy  76 % [68-83] | Sens 96 %  [90 - 99]* | Spec 37 % [24-51]* |  | Accuracy 75 % [67-82] |
| **≥2** | 45 | 4 | 49 | PPV 92% [81-97] | 69 | 9 | 78 | PPV 88 % [81-93] |
| **<2** | 50 | 48 | 98 | NPV 49% [44-54] | 26 | 43 | 69 | NPV 62 % [54-70] |
|  | 95 | 52 | 147 |  | 95 | 52 | 147 |  |
|  | Sens 47 % [37-58] | Spec 92 % [81-98] |  | Accuracy 63 % [55-71] | Sens 73 % [63-81]* | Spec 83 % [70-92] |  | Accuracy 76 %* [68-83] |
| **≥3** | 13 | 0 | 13 | PPV 100% [75-100] | 32 | 1 | 33 | PPV 97 % [82-100] |
| **<3** | 82 | 52 | 134 | NPV 39 % [37-41] | 63 | 51 | 114 | NPV 45 % [41-48] |
|  | 95 | 52 | 147 |  | 95 | 52 | 147 |  |
|  | Sens 14 % [7-22] | Spec 100 %  [93 - 100] |  | Accuracy 44 % [36-53] | Sens 34 % [11-27]* | Spec 98 % [90 - 100] |  | Accuracy 56 %* [48-65] |

**Supplemental Table 3. Dutch RPD cohort: performance of the STAM_3_P and modified STAM3^m^P scores**

NPV=negative predictive value. PPV=positive predictive value. Sens=sensivity. Spec=specificity. *Statistically significant difference (p<0.05) between STAM_3_P vs. STAM3^m^P.

**Supplemental Table 4. US RPD cohort: performance of the STAM_3_P and modified STAM3^m^P scores**

|  | **STAM_3_P** | | | | **STAM3^m^P** | | | |
| --- | --- | --- | --- | --- | --- | --- | --- | --- |
|  | **Treatment-**  **responsive** | **Not  responsive** | **AUC = 0.88** | | **Treatment-**  **responsive** | **Not  responsive** | **AUC = 0.88** | |
| **≥1** | 82 | 26 | 108 | PPV 76 % [67-84] | 83 | 41 | 124 | PPV 67 % [62-71] |
| **<1** | 4 | 43 | 48 | NPV 92 % [79-98] | 3 | 28 | 31 | NPV 90 % [75 -97] |
|  | 86 | 69 | 155 |  | 86 | 69 | 155 |  |
|  | Sens 95 %  [89-99] | Spec 62 %  [49-74] |  | Accuracy 81 % [74-87] | Sens 97 % [90-99] | Spec 41 % [29-53]* |  | Accuracy 72 % [64-79] |
| **≥2** | 53 | 5 | 58 | PPV 91 % [81-97] | 71 | 11 | 82 | PPV 87 % [79-92] |
| **<2** | 33 | 64 | 97 | NPV 66 % [56-75] | 15 | 58 | 73 | NPV 79 % [71-86] |
|  | 86 | 69 | 155 |  | 86 | 69 | 155 |  |
|  | Sens 62 % [51-72] | Spec 93 % [84-98] |  | Accuracy 75 % [68-82] | Sens 83 % [73-90]* | Spec 84 % [73-92]* |  | Accuracy 83 % [76-89] |
| **≥3** | 22 | 0 | 22 | PPV 100 % [85-100] | 38 | 2 | 40 | PPV 95 % [83 - 99 ] |
| **<3** | 64 | 69 | 123 | NPV 52 % [43-61] | 48 | 67 | 115 | NPV 58 % [54 - 63] |
|  | 86 | 69 | 155 |  | 86 | 69 | 155 |  |
|  | Sens 26 % [17-36] | Spec 100 % [95-100] |  | Accuracy 59 % [51-67] | Sens 44 % [33-55]* | Spec 97 % [90 – 100] |  | Accuracy 68 %* [60-75] |

NPV=negative predictive value. PPV=positive predictive value. Sens=sensivity. Spec=specificity. *Statistically significant difference (p<0.05) between STAM_3_P vs. STAM3^m^P.

**Supplemental Table 5.** **Sensitivity of the modified STAM3^m^P score and possible AE criteria across diagnostic categories (US and Dutch RPD Cohort combined)**

| **Etiological diagnostic categories** | **Total (n=302)** | **STAM3^m^P**  **≥1** | **STAM3^m^P**  **≥2** | **STAM3^m^P**  **≥3** | **Possible**  **AE criteria** |
| --- | --- | --- | --- | --- | --- |
| **Treatment-responsive** | 181 (60) | 174 (96) | 140 (77) | 70 (39) | 129 (71) |
| Autoimmune encephalitis | 109 (36) | 108 (99) | 93 (85) | 54 (50) | 100 (92) |
| Inflammatory CNS disorder | 28 (9) | 25 (89) | 23 (82) | 7 (25) | 16 (57) |
| Toxic/metabolic | 16 (5) | 15 (94) | 8 (50) | 2 (13) | 4 (25) |
| Neoplastic | 11 (4) | 11 (100) | 8 (73) | 3 (27) | 3 (27) |
| Primary psychiatric disorder | 8 (3) | 6 (75) | 2 (25) | 0 (0) | 1 (13) |
| Epilepsy (non-inflammatory) | 4 (1) | 4 (100) | 4 (100) | 3 (27) | 2 (50) |
| Vascular | 2 (1) | 2 (100) | 0 (0) | 0 (0) | 0 (0) |
| CNS infection | 3 (1) | 3 (100) | 2 (67) | 1 (33) | 3 (100) |
| **Non-responsive** | 121 (40) | 74 (61) | 20 (17) | 3 (2) | 12 (9) |
| Neurodegenerative diseases | 54 (18) | 24 (44) | 4 (7) | 0 (0) | 4 (7) |
| Creutzfeldt-Jakob disease | 42 (14) | 31 (74) | 10 (24) | 3 (7) | 1 (2) |
| Vascular | 15 (5) | 13 (87) | 4 (27) | 0 (0) | 4 (27) |
| Neoplastic | 2 (1) | 1 (50) | 1 (50) | 3 (27) | 1 (50) |
| Other* | 8 (3) | 5 (63) | 1 (13) | 0 (0) | 2 (25) |

CNS=central nervous system. *6 unknown, 2 leukoencephalopathy

| **Pt.  no** | **Sex; Age (Range)** | **Clinical presentation** | **First line Ancillary testing** | **STAM3^m^P score** | **Clinical**  **impact** | **Final diagnosis** |
| --- | --- | --- | --- | --- | --- | --- |
| 1 | Male;  65-69 | RPD (<3 months) | MRI: bithalamic T2/FLAIR enhancing hyperintense lesion.  CSF: WBC 58/mm^3^  Tumor screening: negative | 2 | Diagnostic: digital subtraction angiography | Dural arteriovenous fistula |
| 2 | Female;  60-65 | RPD (<3 months) | MRI: intraventricular enhancement  CSF: WBC 62/mm^3^  Tumor screening: negative | 2 | Diagnostic: brain biopsy | Primary central nervous system lymphoma |
| 3 | Male;  70-75 | RPD (<3 months) | MRI: normal  CSF: WBC 2/mm^3^  Tumor screening: lung carcinoma | 2 | Diagnostic: autoantibody studies | Anti-NMDARE |
| 4 | Male;  60-65 | RPD (<3 months), aphasia, seizures, R arm dystonia | MRI: T2/FLAIR cortical hyperintensities, restricted diffusion  CSF: WBC 2/mm^3^  Tumor screening: negative | 3 | Diagnostic and therapeutic: empiric immunotherapy and brain biopsy | Primary angiitis of the Central Nervous System |
| 5 | Male;  50-55 | RPD (<3 months), seizures, myoclonus | MRI: T2/FLAIR cortical-subcortical hyperintensities  CSF: WBC 61 L/mm^3^  Tumor screening: negative | 5 | Diagnostic and therapeutic: immunotherapy autoantibody studies | Anti-GABA_A_ receptor  encephalitis |

**Supplemental Table 6.** **Case examples of treatment-responsive RPD demonstrating clinical application of the STAM3^m^P score**

RPD=rapidly progressive dementia. CSF=cerebrospinal fluid. MRI=magnetic resonance imaging. R=right. WBC=white blood cell count

**Supplemental Figure 1. Comparison of Receiver Operating Characteristic (ROC) Area Under the Curve (AUC) of STAM_3_P and modified STAM3^m^P across different rapidly progressive dementia cohorts (Dutch, US and combined)**

**References**

1.	Graus, F., et al., *A clinical approach to diagnosis of autoimmune encephalitis.* Lancet Neurol, 2016. **15**(4): p. 391-404.

2.	Hermann, P., et al., *Biomarkers and diagnostic guidelines for sporadic Creutzfeldt-Jakob disease.* Lancet Neurol, 2021. **20**(3): p. 235-246.
